# Fabry Disease: A Rare Mutation With Common Clinical Presentation

**DOI:** 10.7759/cureus.71924

**Published:** 2024-10-20

**Authors:** Mariana Certal, Elisabete Cerqueira, Cátia Ribeiro, Marta B Santos, Sandra Tavares

**Affiliations:** 1 Internal Medicine, Unidade Local de Saúde de Trás-os-Montes e Alto Douro, Chaves, PRT

**Keywords:** cerebrovascular accident, fabry's disease, mutation, stroke, x-linked disease, α-galactosidase a

## Abstract

Strokes are infrequent in younger adults, making diagnosis of their underlying causes challenging. Fabry disease, a rare genetic condition with a complex and not fully understood pathophysiology, is one potential cause. This report describes a 41-year-old woman with a history of glaucoma, recurrent uveitis, ischemic stroke affecting the posterior circulation, and sensorineural hearing loss. She was admitted to the emergency department with dysarthria, left facial paralysis, and sudden onset left hemiparesis. Imaging revealed an acute ischemic lesion and hypoplasia of the right vertebral artery. Genetic testing for CADASIL (cerebral autosomal dominant arteriopathy with subcortical infarcts and leukoencephalopathy) and MELAS (mitochondrial encephalomyopathy, lactic acidosis, and stroke-like episodes) was negative, but a heterozygous c.937G>T (p.Asp313Tyr) variant in the GLA gene was detected, indicating Fabry disease. This case underscores the importance of early diagnosis, given the potential for treatment with enzyme replacement therapy.

## Introduction

Fabry disease (FD) is a rare, hereditary, X-linked recessive lysosomal storage disorder caused by various mutations in the α-galactosidase A (GLA) gene. These mutations result in a partial or complete deficiency of the enzyme α-galactosidase A (α-Gal A). To date, over 700 mutations in the GLA gene have been identified [1], though the association of some with FD remains debated. The enzyme deficiency causes systemic accumulation of globotriaosylceramide and related glycosphingolipids in plasma and tissue lysosomes, leading to a multi-systemic disease [2].

As an X-linked disease, hemizygous men typically exhibit more pronounced clinical manifestations, while women are often asymptomatic or show milder signs and symptoms. However, in some cases, due to lyonization or random X-inactivation, females may be as severely affected as hemizygous males [3].

FD is a frequently underdiagnosed rare disorder that leads to a range of clinical manifestations, including acroparesthesias, angiokeratomas, hypohidrosis, hearing loss, and corneal dystrophy. Additionally, renal failure, cardiac disease, and cerebrovascular complications have been reported, resulting from the progressive accumulation of lipids in lysosomes within the vascular endothelium [4]. Several studies have also reported both central and peripheral neurological complications, including stroke, which is often the leading cause of hospitalization. In the group of neurological complications, it is important to highlight peripheral neuropathy and autonomic nervous system dysfunctions, such as hypohidrosis and orthostatic hypotension.

The incidence of stroke increases with age and is usually low in young adults, where determining the cause poses a significant challenge, as 20-40% of cases remain undetermined. Although rare, FD is a recognized cause of stroke, and cerebrovascular disease is one of its major manifestations. Many patients experience a stroke prior to receiving an FD diagnosis [4]. The average age of onset for cerebrovascular complications in FD is 32 years, and the cerebral manifestations are classified as large- and small-vessel diseases. Large-vessel strokes are caused by occlusion of major vessels or cardiac embolism, while small-vessel disease commonly appears as subcortical strokes or asymptomatic cortical white matter hyperintensities. Strokes can occur in both circulatory systems. In 56% of cases, the clinical presentation involves vertebrobasilar ischemia, while only 20% affect the anterior circulation. FD is also linked to white matter lesions and vertebrobasilar artery dolichoectasia [5]. Despite the high incidence of cerebrovascular complications in FD, the pathogenic mechanism remains unclear, though it is known to be a multifactorial process.

## Case presentation

We describe a case of a 41-year-old Caucasian woman who was observed in the internal medicine emergency department due to dysarthria, left lip commissure deviation, and sudden onset hemiparesis. On physical examination, she was hemodynamically stable and apyretic, and her cardiac and pulmonary auscultations were normal. On neurological examination, she was alert and orientated, with left facial paralysis, and left hemiparesis. She had a past history of glaucoma, recurrent uveitis, posterior circulation transitory ischemic stroke two years before, and left ear neurosensorial hearing loss. She had a familiar history of hearing loss and decreased visual acuity of her mother and sister, respectively. The patient was prescribed acetylsalicylic acid at a dose of 100 mg/day. She denied the use of oral contraceptives, smoking, drinking, or drug use.

Routine hematological tests were normal, including the coagulation tests and renal function (Table 1).

**Table 1:** Laboratory tests of the patient on admission. aPTT: activated partial thromboplastin time; CRP: C-reactive protein; ESR: erythrocyte sedimentation rate; HbA1c: hemoglobin A1c; HDL: high-density lipoprotein; INR: international normalized ratio; LDH: lactate dehydrogenase; LDL: low-density lipoprotein.

Test	Result	Reference range
Hemoglobin	13.9	12-16 g/dL
Leukocytes	9800	4000-11000/µL
Platelets	287000	150000-400000/µL
ESR	30	0-15 mm/h
CRP	0.54	<0.5 mg/L
Creatinine	0.7	0.6-1.1 mg/dL
Sodium	138	135-147 mEq/L
Potassium	4.5	3.7-5.1 mEq/L
INR	1.03	<1.2
aPTT	30.2	24-35 seg
Total cholesterol	190	<200 mg/dL
HDL	37	>60 mg/dL
LDL	129	<130 mg/dL
Triglycerides	86	<150 mg/dL
HbA1c	5.8	4-6%
Homocysteine	9.0	4.1-13.5 µmol/L

**Figure 1:**
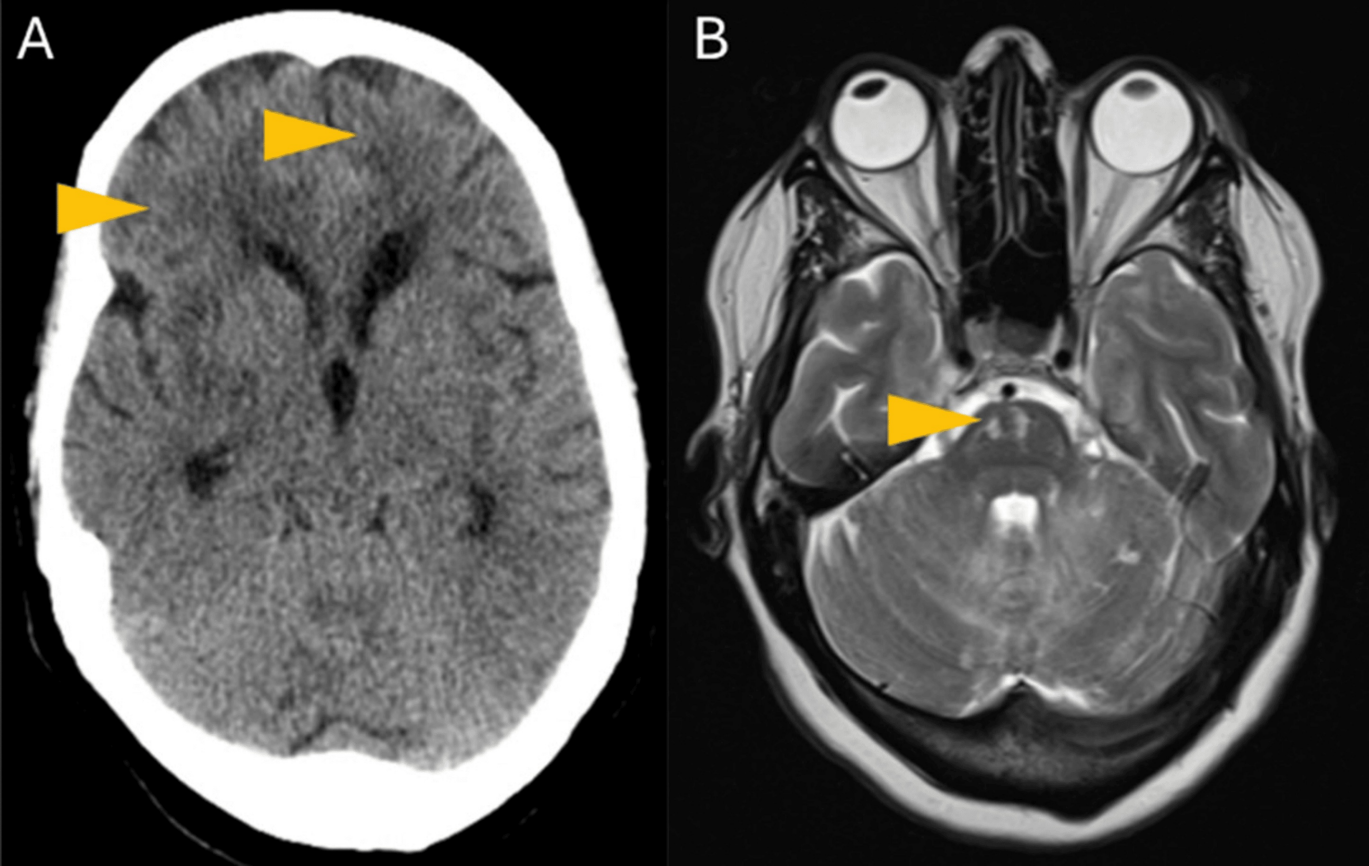
Brain CT and MRI images. A computed tomography (CT) scan in the axial view revealed anterior bulging hypodensities, more evident in the right hemisphere (A). T2-weighted magnetic resonance imaging (MRI) in the axial view showing an acute ischemic injury in the right basilar artery territory (B).

A computed tomography (CT) scan revealed anterior bulging hypodensities, more evident on the right hemisphere, and the brain magnetic resonance imaging (MRI) showed an acute ischemic injury in the right basilar artery territory and right vertebral artery hypoplasia (Figure 1).

**Figure 2:**
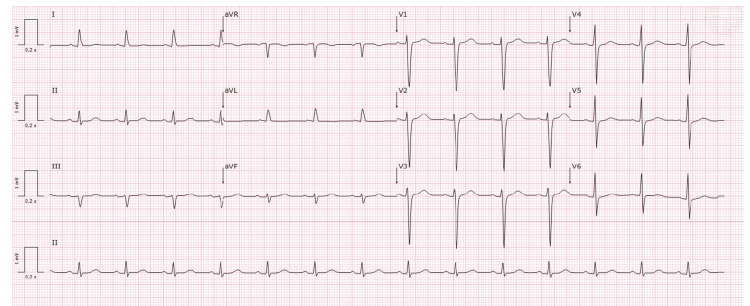
Electrocardiography of the patient. Electrocardiogram showing a sinus rhythm.

**Figure 3:**
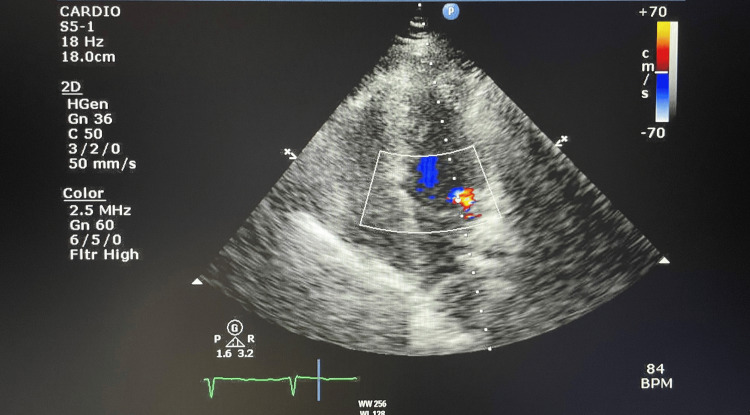
Echocardiography of the patient. Echocardiogram revealing mild mitral regurgitation with slight prolapse movement.

Findings on carotid ultrasonography were normal. Electrocardiography showed sinus rhythm (Figure 2) and echocardiography revealed mild mitral regurgitation with slight prolapse movement (Figure 3).

The remaining study included a negative immunological study, syphilis serology, viral serology, and prothrombotic study (Table 2).

**Table 2:** Serologic tests of the patient. ANAs: antinuclear antibodies; CMV: cytomegalovirus; HCV: hepatitis C virus; HIV: human immunodeficiency virus; Ig: immunoglobulin; RPR: rapid plasma reagin test; TPHA/TPPA/TP: treponema pallidum hemagglutination assay/particle agglutination assay.

Test	Result
Anti-CMV	
IgG	Reactive
IgM	Non-reactive
HBs antigen	Negative
Anti-Hbs	Non-reactive
Anti-HCV	Non-reactive
Anti-HIV	Non-reactive
ANAs	Negative
Anti-β2 glycoprotein	
IgG	Negative
IgM	Negative
Anti-cardiolipin	
IgG	Negative
IgM	Negative
TPHA/TPPA/TP	Negative
RPR	Negative

The cytochemical cerebrospinal fluid was normal and venereal disease research laboratory (VDRL) and Borrelia serology were negative. The genetic tests for CADASIL (cerebral autosomal dominant arteriopathy with subcortical infarcts and leukoencephalopathy) and MELAS (mitochondrial encephalomyopathy, lactic acidosis, and stroke-like episodes) were negative. The genetic test for FD showed a heterozygotic variant of the GLA gene for Fabry (Table 3).

**Table 3:** Genetic tests of the patient. CADASIL: cerebral autosomal dominant arteriopathy with subcortical infarcts and leukoencephalopathy; MELAS: mitochondrial encephalomyopathy, lactic acidosis, and stroke-like episodes.

Test	Result
CADASIL	Negative
Fabry disease	Heterozygous c.937G>T variant (p.Asp313Tyr)
MELAS	Negative

**Figure 4:**
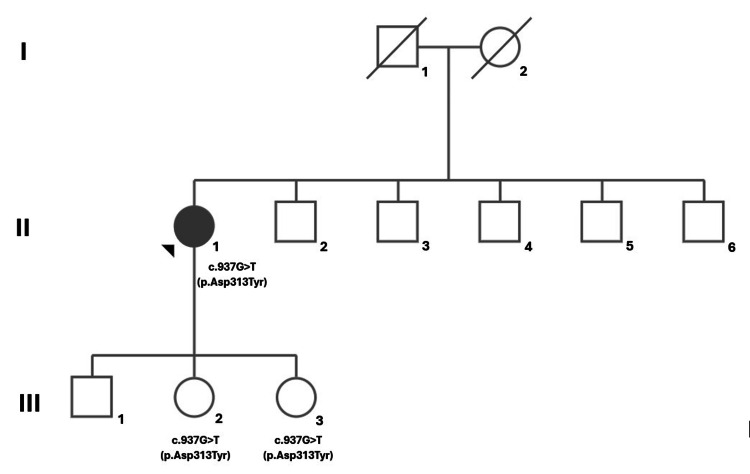
Pedigree of the patient's family. The black symbol indicates patients with Fabry disease phenotype. The proband and patient's daughters (III-2 and III-3) have c.937G>T (p.Asp313Tyr) mutation.

Her plasma α-Gal A and β-galactosidase activity, as well as urinary globotriaosylceramide levels, showed no significant abnormalities. Genetic testing of the GLA gene revealed a heterozygous c.937G>T variant (p.Asp313Tyr). An ophthalmology evaluation was conducted to determine whether the pre-existing eye condition was related to this genetic variant, but the association was ruled out. A family study identified the same mutation in two patient's daughters (Figure 4), though they have not shown any clinical signs of FD to date.

The case was discussed in a multidisciplinary consultation but enzyme replacement therapy (ERT) with beta-galactosidase was not initiated, as there was insufficient evidence at the time to support its benefit in this case. Despite stable renal and cardiac function, the patient's audition and visual acuity declined significantly, corneal opacities developed, and her autonomy diminished as the disease rapidly progressed to an advanced stage.

## Discussion

Although rare, FD often has a poor prognosis, presenting as a stroke, often before diagnosis. Laboratory diagnosis of FD involves the demonstration of enzyme deficiency and enzyme analysis may occasionally help to detect heterozygotes, but is often inconclusive due to random X chromosome inactivation. Diagnostic confirmation is made by demonstrating a genetic mutation in the α-GLA gene; however, there are many controversies in some of them.

The clinical case presented involves a heterozygous c.937G>T (p.Asp313Tyr) mutation in the α-galactosidase A (GLA) gene, which is associated with FD. Historically, the p.Asp313Tyr variant was considered a pseudodeficiency allele, not causing classical Fabry manifestations. Earlier studies suggested that individuals carrying this mutation had reduced alpha-galactosidase A activity without significant clinical consequences, leading some authors to dismiss its role in FD pathology. However, recent studies have started to challenge this view, with emerging evidence suggesting that D313Y is associated with an atypical, late-onset, and predominantly neurologic phenotype [6]. In this case, laboratory findings confirm a heterozygous p.Asp313Tyr mutation, which highlights the need to reconsider this variant's contribution to FD, especially in light of its evolving characterization. Atypical FD cases, particularly those involving cerebrovascular complications like recurrent stroke, are becoming more recognized, and this aligns with findings from other studies that describe neurologic manifestations, such as stroke or transient ischemic attacks, as prominent in patients with this mutation. This points toward an atypical but significant presentation of FD, even in mutations previously considered benign or pseudodeficience.

Cerebral manifestations in patients with FD are categorized into large- and small-vessel diseases [7]. Large-vessel strokes result from occlusion of major intracranial vessels or cardiac embolism, while small-vessel disease, more common in FD patients, typically presents as subcortical strokes or asymptomatic cortical white matter hyperintensities and subcortical infarcts visible in neuroimaging studies [7]. Strokes in FD can occur in both anterior and posterior circulatory systems, affecting cortical and subcortical regions. However, the mechanisms and topography of strokes in FD have not been systematically studied, as research has primarily focused on cryptogenic strokes instead of all stroke types [8]. Additionally, the presence of large artery disease and arrhythmia due to cardiomyopathy in FD patients may distort the observed patterns of infarcts in descriptive studies [8].

There is evidence that the genotype-phenotype correlation does not exist even among members of the same family [9]. In fact, several mutations have been associated with FD and most of them are family-specific, but few occur with sufficient frequency to permit a genotype-phenotype correlation. Even within families, phenotypic heterogeneity is often present, suggesting the possibility of gene-environment interaction [10]. This is particularly true for heterozygous females due to random X-chromosome inactivation, which can cause variable enzyme activity and clinical expression. Some patients may exhibit severe symptoms, while others with the same mutation may remain asymptomatic. The phenotypic variability observed in this patient supports the hypothesis that additional genetic or environmental factors contribute to the clinical presentation of FD. Thus, an exhaustive clinical study of heterozygous c.937G>T gene mutation carriers is essential to define its association with FD.

Current pathophysiological theories suggest that FD-related cerebrovascular disease may arise from multiple mechanisms. These include progressive glycosphingolipid (GL) deposition in vascular endothelial cells, which leads to vessel deformity and occlusion of small arteries. Additionally, cardiogenic embolism, secondary to cardiac involvement such as left ventricular hypertrophy or arrhythmias, is another proposed mechanism of stroke in FD patients [5]. However, none of these mechanisms seem sufficient to fully explain the cerebrovascular complications observed in this patient, suggesting that other genetic or environmental factors may modulate the disease process. In this case, the absence of classical FD manifestations like kidney involvement or severe cardiac pathology raises the possibility that another yet unidentified factor may be interacting with the D313Y mutation to produce the cerebrovascular phenotype.

Given the complexity of FD's pathophysiology and the variability of its clinical presentation, an exhaustive and individualized clinical evaluation is essential for patients with the p.Asp313Tyr variant. Genetic testing alone may not suffice in predicting the disease course, as the interplay between genetic and environmental factors likely contributes significantly to the observed outcomes. Moreover, longitudinal studies and broader genetic screenings are needed to clarify the exact role of the p.Asp313Tyr variant in FD, particularly in the context of cerebrovascular disease.

## Conclusions

This clinical case highlights that heterozygous women can develop severe symptoms of FD, focusing on the rare and controversial D313Y variant of the α-GLA gene, characterized by the c.937G>T mutation, which is now known to play a role in atypical forms of FD. Despite normal alpha-galactosidase A activity, other underlying pathophysiological mechanisms may contribute to disease development. The observed cerebrovascular complications emphasize the importance of recognizing atypical presentations and conducting thorough clinical evaluations. Future research should explore genetic and environmental factors that may influence the phenotype, which is crucial for improving diagnostic accuracy and tailoring treatment strategies.
